# Acupuncture for post-stroke hiccup: an overview of systematic reviews

**DOI:** 10.3389/fneur.2025.1684772

**Published:** 2025-10-23

**Authors:** Xin-xin Liu, Ying-qi Ma, You-zhu Su, Ling-yao Kong, Chen Shen, Jian-ping Liu

**Affiliations:** ^1^Centre for Evidence-Based Chinese Medicine, Beijing University of Chinese Medicine, Beijing, China; ^2^School of Basic Medical Sciences, Guangzhou University of Chinese Medicine, Guangzhou, Guangdong, China; ^3^The National Research Center in Complementary and Alternative Medicine (NAFKAM), Department of Community Medicine, Faculty of Health Science, UiT The Arctic University of Norway, Tromsø, Norway

**Keywords:** acupuncture, post-stroke hiccup, quality of life, methodological quality, quality of evidence, overview of systematic reviews

## Abstract

**Objective:**

This study aimed to describe and summarize the evidence from systematic reviews (SRs) of the efficacy and safety of acupuncture in the treatment of post-stroke hiccup and to provide a reference for clinical practice and future research.

**Methods:**

We conducted a systematic search across eight electronic databases from their inception up to February 2025. This search encompassed systematic reviews focusing on acupuncture for post-stroke hiccup. The methodological quality was assessed using the AMSTAR 2. The corrected coverage area was used to calculate the degree of overlap of the original studies. The evidence quality was assessed using the GRADE approach.

**Results:**

Of the 10 eligible systematic reviews included, the methodological quality was evaluated as 1 (10.00%) low and 9 (90.00%) very low quality. The corrected coverage area of the preliminary studies was moderate or high overlap. Acupuncture may alleviate hiccups symptoms and improve the quality of life (certainty: moderate to very low). Safety data were limited and insufficient to draw firm conclusions.

**Conclusion:**

Acupuncture may be a potential intervention for the treatment of a post-stroke hiccup. These results should be interpreted with caution due to the methodological shortcomings and low evidence quality observed in current systematic reviews. There is an urgent need for more well-designed, high-quality randomized controlled trials.

## 1 Introduction

Hiccups are a common yet often overlooked complication following stroke (1). Research indicates that the frequency of post-stroke hiccups varies among different stroke types, with brainstem strokes and medullary damage showing a higher prevalence of symptoms (2). This is due to the close relationship between hiccup occurrence and the neural centers in the brainstem, which regulate respiratory and digestive reflexes. When these regions are affected by stroke, patients are more prone to experiencing recurrent hiccup episodes. Epidemiological studies reveal that the incidence of post-stroke hiccups varies among different reports, with some estimating rates between 8 and 15% (3, 4). In cases of severe stroke or brainstem involvement, the incidence could reach 20% or higher. Post-stroke hiccups are more commonly observed in males and their incidence tends to increase with age, mirroring higher stroke rates in older populations (5). Additionally, individuals with a history of gastrointestinal disorders are more prone to experiencing hiccups. These recurring or intermittent hiccups not only significantly affect the patient’s quality of life but may further impair respiratory and neurological functions, worsening the overall clinical prognosis (6). Contemporary medical studies suggest that post-stroke hiccups may be associated with central nervous system injury, vagus nerve dysfunction, and gastrointestinal reflex disturbances. However, conventional drug therapies for this condition often yield limited results and come with various adverse effects (7, 8).

Sleep disturbances and emotional problems are frequently encountered after a post-stroke hiccup. The causes, duration, and progression of these issues are generally regarded as multifactorial, involving not only brain damage related to the stroke but also various environmental, psychological, and other contributing factors (9). A growing body of evidence links these disturbances to an elevated risk of stroke recurrence and poorer prognosis (10, 11). On the other hand, there is also evidence suggesting that adequate sleep and a positive mental state can enhance neuroplasticity and facilitate functional recovery after a stroke (12, 13). Nevertheless, current treatments remain largely ineffective, presenting a significant challenge. An increasing number of studies shows that both traditional and supplementary therapies have a positive effect on improving these complications (14, 15).

Acupuncture, a key component of traditional Chinese medicine, has a rich history and extensive use in addressing post-stroke complications (16). Through its capacity to regulate meridians, balance qi and blood flow, and support nerve regulation and visceral recovery, acupuncture has gained recognition as an effective complementary approach for managing post-stroke hiccups. Over the past 10 years, numerous systematic reviews (SRs) have examined this topic. This study aims to conduct an overview of SRs to evaluate the validity and reliability of previous findings regarding the efficacy of acupuncture in post-stroke hiccup management. In this overview, we also applied relatively novel systematic review tools, such as the Assessment of Multiple Systematic Reviews (AMSTAR 2) and the Grading of Recommendations Assessment, Development, and Evaluation (GRADE) approach. This overview aims to provide a clearer picture of the role of acupuncture in the treatment of post-stroke hiccup and a more nuanced understanding of the certainty and strength of the available evidence.

Despite the increasing number of SRs addressing acupuncture for post-stroke hiccup, most of them are characterized by methodological shortcomings, inconsistent conclusions, and high overlap of primary trials. Clinicians and policymakers therefore face uncertainty when interpreting the current evidence base. An overview of SRs allows for a comprehensive reappraisal of these reviews by using structured tools such as AMSTAR 2, GRADE, and corrected coverage area (CCA), which can clarify the strength and limitations of existing findings. This approach not only provides a consolidated evidence landscape but also identifies critical gaps for future high-quality trials.

## 2 Methods

### 2.1 Literature retrieval

We systematically searched eight databases, including four English databases: Web of Science, Cochrane Library, PubMed, and EMBASE, and four Chinese databases: CNKI, Wanfang, VIP, and SinoMed from inception to February 2025, without any language restriction. The term includes, but is not limited to, the following contents: post-stroke hiccup, acupuncture, systematic review, and meta-analysis. Detailed search strategies for each database, including the exact terms and limits, are provided in Supplementary Material 1.

### 2.2 Eligibility criteria

(1) Participants: The participants included patients with a confirmed diagnosis of stroke. Stroke was diagnosed according to the 2013 AHA/ASA updated definition (17). Hiccups were diagnosed according to the relevant criteria outlined in the Practical Manual of the Latest Clinical Diagnostic and Therapeutic Techniques in Digestive System Diseases (18), which define persistent hiccups as episodes lasting more than 48 h. The hiccup sounds may vary in pitch and can pause for 30 to 60 min before recurring. In severe cases, hiccups may occur continuously and persist throughout the day and night. There were no restrictions on age, race, or sex.

(2) Interventions: The treatment group underwent standard forms of acupuncture or a combination with other treatments, irrespective of acupoint selection, treatment frequency, or duration.

(3) Comparisons: The control group utilized the conventional Western medicine, such as baclofen, metoclopramide, and anisodamine. Both groups could receive equivalent fundamental treatment.

(4) Outcomes: ① Primary outcomes: hiccup symptom scores; ② secondary outcomes: clinical efficiency and quality of life (sleep scores, dietary scores, and psychological scores); and ③ safety outcomes: adverse events.

(5) Study design: All systematic reviews (SRs) on acupuncture for the treatment of post-stroke hiccup were included. Registration protocols, map reviews, scoping reviews, umbrella reviews, or network meta-analyses were excluded.

### 2.3 Study selection and data extraction

We imported all retrieved literature citations into EndNote 20.0, and two researchers (Xin-Xin Liu and Ying-Qi Ma) independently screened all the literature. The eligible literature was screened and checked for full text. Literature that met the eligibility criteria was identified. Any differences in discussions or requests were solved by the third researcher (Jian-Ping Liu). We used Microsoft Office Excel 2019 to independently extract and cross-check data from the included studies. Extracting information such as ① characteristics of the study: the first author, year of publication, searching databases, country and area, languages, the number of randomized controlled trials (RCTs), and sample size; ② methodological characteristics: participants, interventions, comparisons, outcomes, and quality of RCT assessment tool; and ③ the main conclusions of the SRs. When overlapping SRs included similar sets of primary RCTs, we adopted a prioritization strategy. Preference was given to the more recent reviews that included a larger number of trials, applied more comprehensive search strategies, or demonstrated higher methodological quality (e.g., protocol registration, double screening, or detailed risk-of-bias assessment). To further quantify the degree of overlap among SRs, we constructed a citation matrix and calculated the corrected covered area (CCA). This allowed us to transparently assess the extent of duplication and interpret findings with caution when overlap was substantial. Corresponding authors were contacted by email if any data were missing or incomplete. Any disagreements were resolved by the introduction of the third researcher (Jian-Ping Liu).

### 2.4 Methodological quality evaluation and assessment of risk of bias

We used AMSTAR 2 to assess the methodological quality of the included literature. It consists of research questions, review design, search strategy, study selection, data extraction, rationale for excluding studies, description of included studies, risk of bias, funding sources, meta-analysis, heterogeneity, publication bias, and conflict of interest. Two researchers (Xin-Xin Liu and You-Zhu Su) independently assessed the methodological quality and risk of bias of SRs. During this process, any disagreements were resolved through discussion or by requesting guidance from the 3rd researcher (Jian-ping Liu).

### 2.5 Assessment of overlap in SRs

To recognize the possible risk of bias in the included SRs due to overlapping primary studies, we generated a citation matrix (Supplementary Material 2) and calculated the corrected coverage area (CCA) (19) using Microsoft Office Excel 2019.


$$\mathrm{CCA}=\frac{N-r}{\mathrm{rc}-r}$$

where *N* is the number of publications, r is the number of RCTs, and c is the number of SRs. A CCA value of ≤ 5 indicates mild overlap, 6–10 indicates moderate overlap, 11–15 indicates high overlap, and ≥15 indicates very high overlap.

### 2.6 Summary of the evidence and evaluation of the quality of evidence

For the quantitative synthesis, we reported the effect sizes in SRs: risk ratio (RR), mean deviation (MD) or standardized mean difference (SMD), corresponding 95% CI, and main findings. The quality of evidence was evaluated based on the GRADE methodology. When the authors of the study did not report the grading of the evidence, we re-evaluated it. There were five dimensions considered: limitations (risk of bias), indirectness, inconsistency, imprecision (small number of studies and/or participants), and publication bias. The quality of evidence for each outcome: very low (true effect is likely to be substantially different from the estimated effect), low (true effect may be substantially different from the estimated effect), moderate (the true effect is likely to be close to the estimated effect), and high (the true effect lies close to that of the estimated effect). The following judging criteria were used: no degradation in any of the five dimensions was considered high; a decrease in quality of one level was considered moderate evidence; a decrease in quality of two levels was considered low; a decrease in quality of three or more levels was considered very low. Two researchers (Xin-Xin Liu, Ling-Yao Kong) independently assessed the quality of the evidence in the reviews. Any disagreements in their assessments were resolved through discussion with the third researcher (Jian-Ping Liu).

## 3 Results

**Figure 1:**
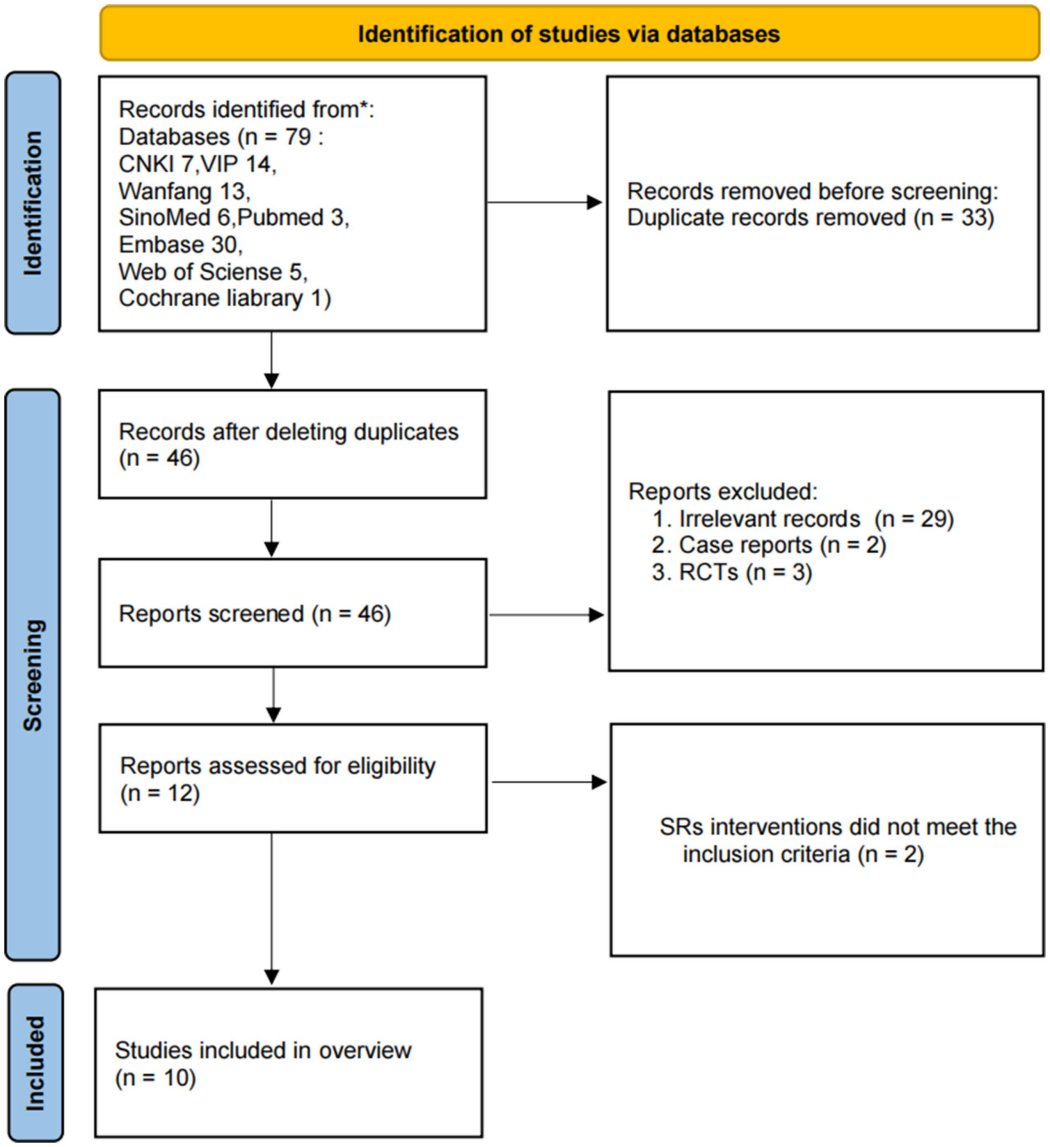
Flowchart of literature screening.

A total of 79 literature articles were retrieved, and 46 were included after de-duplication. Articles that did not meet the eligibility criteria were excluded in the first stage of screening titles and abstracts. Ten SRs (20–29) were included in the review after reading the full text. The screening process is shown in Figure 1.

### 3.1 Characteristics of included SRs

A total of 10 SRs were included, which were published in 2010–2023. Among them, eight were published in Chinese and two (20, 25) in English. Chinese researchers authored the majority of these studies, while the remaining originated in America. The number of randomized controlled trials (RCTs) included in each SR ranged from 3 to 34, with corresponding sample sizes ranging from 143 to 2,450. In the methodological quality assessment of RCTs in SRs, 10 (100.00%) used the Cochrane Collaboration Risk of Bias Assessment Tool (RoB1). Meta-analysis was applied to all SRs. Interventions involved acupuncture or as adjunct therapy. The duration of the intervention ranged from 3 to 28 days. Outcomes measured mainly included clinical efficiency, hiccup symptom scores, incidence of adverse events, and dietary scores. In terms of results and conclusions, all SRs showed that acupuncture improves clinical efficacy, hiccup symptoms, and quality of life. The basic characteristics of the included studies are shown in Table 1.

**Table 1:** Details of characteristics of included SRs.

No.	Study ID	Searching duration	Searching databases	Disease type	No. of included Studies (patients)	acupuncture intervention	Control intervention	Duration	Follow-up	Applied assessment tools	Outcomes	Main conclusions
1	Wang et al. (2023) (20)	Database inception until 2022.02.01	PubMed, Cochrane Library, EMBASE, WOS, Sinomed, WanFang, VIP, ClinicalTrials.gov, Chinese Clinical Trial Registry	Post-stroke hiccup	18 (1416)	Acupuncture; Ear acupuncture; Acupoint sticking	Metoclopramide	4–28 d	No	ROB1	① Clinical efficiency; ② Hiccup Symptom Scores; ③ Life Quality Scores; ④ Adverse Reaction; ⑤ SAS (Self-Rating Anxiety Scale)	Acupuncture could prove effective in post-stroke hiccup treatment.
2	Zhang et al. (2022) (21)	database inception until 2019.02	PubMed, Embase, Cochrane library, CNKI, WanFang, SinoMed,	post-stroke hiccup	19 (1252)	Acupuncture; Electroacupuncture	Anisodamine; Baclofen; Chlorpromazine; Sodium valproate	3–14 d	No	ROB1	① Clinical efficiency;② Hiccup symptom scores;③ Sleep scores;④ Dietary scores;⑤ Psychological scores;⑥Incidence of adverse events;	Acupuncture alone or plus Western medicine treatment can improve clinical efficacy, improve hiccup symptom scores, improve sleep, diet, and mental scores, and do not increase the occurrence of adverse reactions.
3	Zhao (2021) (22)	Database inception until 2020.12	PubMed, Web of Science, Cochrane Library, Chinese Medical Dictionary, National Newspaper Index, CNKI, Wanfang, VIP	post-stroke hiccup	5 (314)	Acupuncture	Chlorpromazine	6–14 d	No	ROB1	① Clinical efficiency;② Hiccup symptom scores;③ Sleep scores;④ Dietary scores;⑤ Psychological scores	Acupuncture treatment for post stroke hiccup patients compared to chlorpromazine has advantages in hiccup symptom scores, effective rate, dietary, sleep and psychiatric scores.
4	Chen et al. (2020) (23)	database inception until 2018.11	PubMed, WOS, Cochrane Library, CNKI, WANGFANG, VIP, Sinomed	post-stroke hiccup	21 (1393)	Acupuncture, Ear acupoint pressing, Electroacupuncture, Scalp acupuncture, Ear acupuncture, Body acupuncture, Acupoint injection, Acupoint pressing	Anisodamine; Baclofen; Chlorpromazine; Sodium valproate	4–14 d	No	ROB1	① Clinical efficiency; ② Cure rate; ③ Dietary scores; ④ Sleep scores; ⑤ Spirit points; ⑥ Recurrence rate	Acupuncture intervention can effectively improve the total effective rate and cure rate of intractable hiccup after stroke, and subgroup analysis suggests that acupuncture can improve the total effective rate of intractable hiccup after stroke; Acupuncture therapy is effective in improving indicators such as hiccup symptom scores, mental state scores, dietary scores, and sleep scores. However, there is insufficient evidence to demonstrate the safety and effectiveness of acupuncture therapy in reducing recurrence.
5	Zhang et al. (2019) (24)	database inception until 2018.10	PubMed, Cochran library, CNKI, WanFang, VIP, Sinomed	post-stroke hiccup	9 (626)	Acupuncture, electroacupuncture	Baclofen, Metoclopramide, Chlorpromazine, Anisodamine, Atropine, Ritalin	3–24 d	Yes	ROB1	① Clinical efficiency; ② Wada drinking water test; ③ hiccup symptom scores; ④ SAS	The clinical efficacy of acupuncture treatment for post stroke hiccup is superior to other control methods, but the quality of the literature included in the current study is relatively low, with a small sample size and insufficient evidence
6	Yue et al. (2017) (25)	database inception until 2015.06	Medline, Embase, Cochrane Central, CENTRAL, CINAHL, Sinomed, CNKI, CAJ, VIP, Wanfang	post-stroke hiccup	5 (259)	Electroacupuncture, Manual acupuncture;	Baclofen, Sodium valproate, Intramuscular anisodamine Hydrobromide, Atropine	7–15 d	No	ROB1	① Clinical efficiency	Acupuncture is effective only when used as an adjunctive intervention; however, the small sample size and poor methodological quality of the included trials prevent us from drawing any firm conclusions.
7	Liu (2015) (26)	database inception until 2014.12	PubMed, Cochrane Library, OVID, Sinomed, WanFang, VIP, CNKI	post-stroke hiccup	34 (2450)	Acupuncture, Acupoint injection, Acupuncture + ear Points, ear points, Electroacupuncture, Acupuncture+ acupoint injection, Electroacupuncture+ acupoint injection	Western medicine therapy	NA	No	ROB1	① Clinical efficiency;② Hiccup symptom scores; ③Traditional Chinese Medicine syndrome integral	Acupuncture has certain efficacy and advantages in treating post stroke hiccup in terms of total effective rate, hiccup symptom scores, TCM syndrome scores and safety. However, due to less research included and limited evidence, more high-quality, multi center, large sample randomized trials are needed
8	He et al. (2013) (27)	database inception until 2012	Cochrane Library, JBI, MEDLINE, Embase, Sinomed, CNKI, VIP, WanFang	post-stroke hiccup	9 (614)	Acupuncture	Metoclopramide; Intramuscular Injection of scopolamine; Chlorpromazine Hydrochloride; Promethazine Hydrochloride	NA	No	ROB1	① Clinical efficiency	Acupuncture is more effective than traditional Western medicine therapy and has no adverse effects
9	Zhu et al. (2011) (28)	database inception until 2010.06	PubMed, Cochrane Library, CNKI, VIP, WanFang	post-stroke hiccup	3 (143)	Acupuncture, Body acupuncture, Ear acupuncture	Conventional therapy	3–20 d	Yes	ROB1	① The mortality rate end of the follow-up period (at least 3 months);② effective rate end of the follow-up period (at least 3 months)	Acupuncture has a short-term trend of improving hiccup after stroke, and there are no adverse reactions. Due to the low quality and limited number of cases in all three included studies, and the observation follow-up period being less than 3 months, it is currently not possible to draw a definite conclusion on their long-term efficacy, and the existing evidence is insufficient to recommend acupuncture as a routine treatment for post-stroke hiccup.
10	Liu et al. (2010) (29)	database inception until 2008.12	CNKI, Sinomed, VIP, WanFang	post-stroke hiccup	6 (497)	Acupuncture, Acupoint injection	Diazepam, Atropine, Chlorpromazine, Diclofenac, Metoclopramide	5–10 d	No	ROB1	① Clinical efficiency; ② Adverse reactions	Acupuncture is safe and effective in treating hiccups after stroke, and is superior to conventional Western medicine.

### 3.2 Methodological quality and risk of bias of SRs

**Figure 2:**
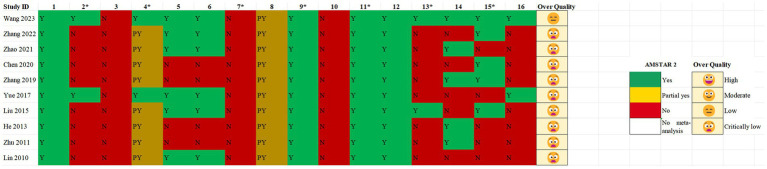
AMSTAR 2 checklist.

Of the 10 SRs, 1 (20) (10%) was of low quality, while 9 (90.00%) were of very low quality. All SRs (100%) reported review questions and eligibility criteria following the patient, intervention, comparison, outcome, and study design (PICOS) principles. Only two SRs (20%) (20, 25) pre-registered their protocol. All reported SRs included RCTs, but their study designs were not explained, and the search strategy was not comprehensive. Four studies (23, 24, 27, 28) did not have two people independently conduct literature screening and data extraction. A list of excluded literature and reasons for exclusion was not provided for all SRs. Ten (100.00%) SRs described the characteristics of some of the included RCTs according to PICO principles. All SRs used appropriate tools to assess the risk of bias in the included studies. All SRs (85.00%) conducted meta-analysis, used appropriate statistical methods, and assessed the publication bias. In interpreting and discussing the results, 10 (100.00%) SRs considered the risk of bias in the included RCTs. Five (20, 21, 23, 24, 26)(50.00%) SRs discussed the possible impact of publication bias on their results. Five (20, 22, 24, 27, 28) SRs (50.00%) discussed the source of heterogeneity. Two (20, 25) (20.00%) SRs mentioned conflicts of interest (see Figure 2).

### 3.3 Overlap between major studies

The CCA was highly or very highly overlapping on each of the outcome measures: clinical efficiency (7.67%), dietary scores (5.55%), hiccup symptom scores (10.00%), psychology scores (11.11%), and sleep scores (11.11%). This meant that several major RCTs were included in duplicate in these SRs (Supplementary Material 2).

### 3.4 GRADE assessment and evidence map

The GRADE assessment of the effects of acupuncture on six outcomes (Table 2).

**Table 2:** Summary of findings of included SRs.

Outcomes	Relative effect (95% CI); heterogeneity	Comparison	No. of RCTs (participants)	Evidence certainty (GRADE)	Notes and comments	SRs	Effect evaluation
Clinical effective	RR 1.27 [1.21,1.33]; *I*^2^ = 9%	Acupuncture vs. Metoclopramide	16 (1206)	MODERATE	1	Wang et al. (2023) (20)	Large
	RR 1.28 [1.13,1.44]; *I*^2^ = 0%	Acupuncture + Chlorpromazine vs. Chlorpromazine	3 (226)	LOW	1,3	Zhang et al. (2022) (21)	Large
	RR 1.23 [1.12,1.26]; *I*^2^ = 69%	Acupuncture vs. Chlorpromazine	15 (926)	LOW	1,2		Large
	RR 1.28[1.14,1.44]; *I*^2^ = 0%	Acupuncture vs. Chlorpromazine	5 (314)	MODERATE	1	Zhao (2021) (22)	Large
	RR 1.19 [1.08,1.30]; *I*^2^ = 79%	Acupuncture vs. WM (Baclofen、Metoclopramide、Anisodamine)	5 (343)	VERY LOW	1,2,3	Chen et al. (2020) (23)	Large
	OR 5.51 [3.22,9.43]; *I*^2^ = 14%	Acupuncture vs. Chlorpromazine	9 (626)	MODERATE	1	Zhang et al. (2019) (24)	Moderate
	RR 1.59 [1.16,1.29]; *I*^2^ = 0%	Acupuncture + WM vs. WM (Baclofen、Sodium valproate、Anisodamine)	3 (136)	Low	1,3	Yue et al. (2017) (25)	Large
	RR 1.40 [0.79,2.47]; *I*^2^ = 65%	Acupuncture vs. Baclofen	2 (123)	Very Low	1,2,3,4		Trivial
	RR 1.33 [1.22,1.45]; *I*^2^ = 63%	Acupuncture vs. WM	10 (547)	Low	1,2	Liu (2015) (26)	Large
	RR 1.31 [1.15,1.49]; *I*^2^ = 0%	Acupuncture + WM vs. WM	4 (216)	Low	1,3		Large
	RR 1.48 [0.96,2.28]; *I*^2^ = 85%	Acupoint injection vs. intramuscular injection	3 (170)	Very Low	1,2,3,4		Trivial
	RR 1.14 [1.07,1.22]; *I*^2^ = 0%	Acupoint injection + Electroacupuncture vs. Electroacupuncture	2 (360)	MODERATE	1		Large
	RR 1.46 [1.14,1.87]; *I*^2^ = 42%	Ear acupuncture vs. Baclofen	2 (97)	Low	1,3		Large
	OR 9.09 [5.31,15.54]; *I*^2^ = 0%	Acupuncture vs. WM (Anisodamine、Chlorpromazine、Metoclopramide)	9 (614)	MODERATE	1	He et al. (2013) (27)	Moderate
	OR 9.05 [3.17,25.82]; *I*^2^ = 0%	Acupuncture vs. WM	3 (143)	Low	1,3	Zhu et al. (2011) (28)	Moderate
	RR 2.82 [2.08,3.83]; *I*^2^ = 82%	Acupuncture vs. Chlorpromazine	2 (270)	Very Low	1,2,3	Liu et al. (2010) (29)	Large
	RR 1.51 [1.08,2.13]; *I*^2^ = 51%	Acupuncture vs. Baclofen	2 (106)	Very Low	1,2,3		Large
	RR 2.55 [1.88,3.45]; *I*^2^ = 85%	Acupoint injection vs. Chlorpromazine	2 (220)	Very Low	1,2,3		Large
Hiccup symptom scores	MD −1.28[−1.64,-0.93]; *I*^2^ = 97%	Acupuncture vs. Metoclopramide	12 (973)	Low	1,2	Wang et al. (2023) (20)	Large
	MD −1.41[−1.72,-1.00]; *I*^2^ = 0%	Acupuncture vs. Chlorpromazine	7 (491)	MODERATE	1	Zhang et al. (2022) (21)	Large
	MD −1.52[−1.94,-1.09]; *I*^2^ = 0%	Acupuncture vs. Chlorpromazine	2 (182)	Low	1,3	Zhao (2021) (22)	Large
	MD −0.06[−0.91,0.80]; *I*^2^ = 0%	Acupuncture vs. WM (ValproateSodium(1d))	2 (88)	Very Low	1,3,4	Liu (2015) (26)	Trivial
	MD −1.40[−0.75,−2.05]; *I*^2^ = 26%	Acupuncture vs. WM (ValproateSodium(3d))	4 (188)	Low	1,3		Large
	MD −2.02[−1.48,−2.56]; *I*^2^ = 0%	Acupuncture vs. WM (ValproateSodium(5d))	4 (188)	Low	1,3		Large
	MD −1.98[−1.44,−2.51]; *I*^2^ = 0%	Acupuncture vs. WM (ValproateSodium(7d))	4 (186)	Low	1,3		Large
Sleep scores	MD 0.44 [0.16,0.72]; *I*^2^ = 0%	Acupuncture vs. WM (Baclofen, Anisodamine)	3 (184)	Low	13	Zhang et al. (2022) (21)	Large
	MD 0.78 [0.48,1.07]; *I*^2^ = 0%	Acupuncture vs. Anisodamine	2 (182)	Low	1,3	Zhao (2021) (22)	Large
	SMD 1.14 [0.60,1.68]; *I*^2^ = 82%	Acupuncture vs. WM (Anisodamine, Metoclopramide, Baclofen)	5 (360)	Very Low	1,2,3	Chen et al. (2020) (23)	Large
Dietary scores	MD 1.39 [0.37,3.16]; *I*^2^ = 97%	Acupuncture vs. WM (Baclofen, Anisodamine)	3 (184)	Very Low	1,2,3	Zhang et al. (2022) (21)	Large
	MD 0.59 [0.29,0.88]; *I*^2^ = 0%	Acupuncture vs. Anisodamine	3 (180)	Low	1,3	Zhao (2021) (22)	Large
	SMD 1.00 [0.43,1.58]; *I*^2^ = 78%	Acupuncture vs. WM (Anisodamine, Baclofen)	4 (240)	Very Low	1,2,3	Chen et al. (2020) (23)	Large
Psychology scores	MD 0.58 [0.22,0.93]; *I*^2^ = 34%	Acupuncture vs. WM (Baclofen, Anisodamine)	3 (184)	Low	1,3	Zhang et al. (2022) (21)	Large
	MD 0.53 [0.26,0.80]; *I*^2^ = 0%	Acupuncture vs. Anisodamine	3 (180)	Low	1,3	Zhao (2021) (22)	Large
	SMD 1.02 [0.61,1.43]; *I*^2^ = 70%	Acupuncture vs. WM (Anisodamine、Metoclopramide、Baclofen)	5 (360)	Very Low	1,2,3	Chen et al. (2020) (23)	Large
Incidence events rate	RR 0.45 [0.16,1.25]; *I*^2^ = 0%	Acupuncture vs. Metoclopramide	2 (156)	Low	1,3	Wang et al. (2023) (20)	Large

MD, mean difference; SMD, standardized mean difference; RR, relative risk; WM, Western medicine.1. Have not implemented allocation concealment and blinding methods;2. High heterogeneity and little overlapping confidence intervals;3. Sample size < 400;4. Merge effect values across invalid lines.

### 3.5 Summary of findings from included SRs

The most commonly reported outcome measures are clinical efficiency, hiccup symptom scores, sleep scores, dietary scores, psychological scores, and incidence of adverse events.

#### 3.5.1 Improvement of hiccup symptom scores

Five studies (20, 22, 24, 27, 28) reported the effect of acupuncture on hiccup symptom scores with a moderate overlap (CCA = 10.00%) among the original studies, and a total of 20 major RCTs were included. The five studies found that acupuncture, or in combination with conventional treatment, improved hiccup symptoms compared to conventional treatment (certainty: moderate to very low). However, one study (29) evaluated acupuncture on day 1 with no statistically significant difference in results (MD -0.06 [−0.91, 0.80]) (certainty: very low) (Table 2).

#### 3.5.2 Improvement of clinical efficiency

The effect of acupuncture on clinical effectiveness was reported in 10 studies with a moderate overlap between the original studies involved (CCA = 7.67%), and a total of 55 major RCTs were included. Of these, some studies reported that acupuncture or adjunctive conventional therapy increased clinical effectiveness compared with conventional therapy (certainty: moderate to very low) (Table 2).

#### 3.5.3 Improvement of sleep scores

Three SRs (21–23) reported the effect of acupuncture on sleep in patients with post-stroke hiccup. The original studies involved were highly overlapping (CCA = 11.11%) and included a total of nine major RCTs. Acupuncture, or in combination with conventional treatment, improved sleep quality compared with conventional treatment (certainty: low to very low) (Table 2).

#### 3.5.4 Improvement of dietary scores

Three SRs (21–23) reported the effect of acupuncture on diet in patients with post-stroke ergogenicity. The original studies involved were moderately overlapping (CCA = 5.55%) and included a total of nine major RCTs. There was a statistically significant difference compared with conventional treatment. It was shown that acupuncture, or in combination with conventional treatment, improves diet (certainty: low to very low) (Table 2).

#### 3.5.5 Improvement of the psychology score

Three SRs (21–23) reported the effect of acupuncture on the psychological status of patients with post-stroke hiccups. The original studies involved were highly overlapping (CCA = 11.11%) and included a total of nine major RCTs. There was a statistically significant difference compared with conventional treatment. It was shown that acupuncture, or in combination with conventional treatment, was able to modify the psychological status of patients (certainty: low to very low) (Table 2).

#### 3.5.6 Improvement of the incidence of adverse events

One SR (20) reported the effect of acupuncture on the incidence of adverse events. The original studies involved were mildly overlapping (CCA = 0.00%). Statistically significant difference was RR 0.45 [0.16, 1.25] compared with conventional treatment (certainty: low). The remaining two SRs (21, 29) were not quantitatively synthesized, but had fewer adverse events than the control group, as detailed in Tables 2, 3.

**Table 3:** Incidence of adverse events.

Study ID	Adverse event category	Experimental group	Control group
Wang et al. (2023) (20)	Hematoma	1 (0.64%)	0 (0.00%)
	Dizziness	0 (0.00%)	1 (0.64%)
	Pain	2 (1.28%)	0 (0.00%)
	Nausea	0 (0.00%)	8 (5.13%)
	Sleepiness	0 (0.00%)	7 (4.49%)
Zhang et al. (2022) (21)	Hematoma	2 (0.82%)	0 (0.00%)
	Pain	2 (0.82%)	0 (0.00%)
	Nausea	0 (0.00%)	5 (2.04%)
	Sleepiness	1 (0.41%)	7 (2.86%)
	Diarrhea	0 (0.00%)	4 (1.63%)
	Dry mouth	2 (0.82%)	1 (0.41%)
	Blurred vision	1 (0.41%)	2 (0.82%)
Zhao (2021) (22)	NA	NA	NA
Chen et al. (2020) (23)	NA	NA	NA
Zhang et al. (2019) (24)	NA	NA	NA
Yue et al. (2017) (25)	NA	NA	NA
Liu (2015) (26)	NA	NA	NA
He et al. (2013) (27)	NA	NA	NA
Zhu et al. (2011) (28)	NA	NA	NA
Liu et al. (2010) (29)	Disorders of consciousness	0 (0.00%)	25 (100.00%)

## 4 Discussion

### 4.1 Summary of main findings

This review summarizes the effectiveness and safety of acupuncture in patients with post-stroke eruption. Only one (29) SR had unrelieved symptoms of hiccups on day 1 of acupuncture intervention, whereas the remaining nine reached positive conclusions regarding different outcomes. Methodological quality assessment of the low quality reported by most studies (9/10) was mainly because most SRs were not preregistered, did not provide a rationale for exclusion from the literature, and did not consider the risk of bias for inclusion in the study. For each outcome indicator, CCA showed moderate to high overlap, which may indicate duplicate reviews. The GRADE assessment rated the majority of evidence as low or very low quality.

Compared with the earlier systematic review and meta-analysis conducted by Yue et al. (25), which synthesized only five small RCTs published before mid-2015, our study represents an overview of systematic reviews. By incorporating 10 SRs published up to 2023, we not only summarized a larger body of evidence but also evaluated a broader range of outcomes, such as sleep, dietary, psychological status, and safety indicators. In addition, we conducted a more comprehensive appraisal by applying established methodological frameworks such as AMSTAR 2, GRADE, and corrected coverage area (CCA) to assess review quality, evidence certainty, and study overlap. These methodological and temporal advances provide a more rigorous and integrative perspective than prior reviews and thereby offer added value for clinical applications and guiding future research planning.

#### 4.1.1 Methodological quality

This overview included 10 SRs published from 2010 to 2023. The overall study quality was generally low. Only a few SRs discussed the risk of bias and its possible impact on their results. Most studies were considered to have an overall high risk of bias, mainly due to incomplete database search strategies, no report of double extraction or double evaluation, unclear data collection methods, high heterogeneity among studies, unclear interpretation of heterogeneity, or high risk of bias from the main study.

The methodological quality needs to be strengthened: (1) retrieval must be comprehensive, which should include the clinical trials registry platform as well as the gray literature to mitigate retrieval bias; (2) studies must provide comprehensive details about participants, interventions, results, research design, follow-up procedures, and study locations; (3) a clear list of excluded studies and the reasons for exclusion should be provided; (4) the rational use of funnel plots or some other tools for assessing publication bias is recommended; (5) to ensure the transparency of the study, the study should be registered in an international pre-registration database in advance, and the study plan should be outlined; (6) researchers should provide a reasoned explanation for high heterogeneity and publication bias in their discussions; and (7) researchers should mention conflicts of interest in the manuscript. The above illustrates the use of acupuncture in the treatment of post-stroke hiccup. SR quality is low, and there is still much room for improvement.

#### 4.1.2 Evidence of acupuncture for post-stroke hiccup

##### 4.1.2.1 Improvement in symptoms of hiccups

Recent findings suggest that acupuncture significantly improves the symptoms of hiccups in patients with post-stroke hiccups and improves clinical outcomes. However, one study found that acupuncture should be used for a minimum of 3 days to treat the symptoms of hiccups. None of the remaining studies mentioned the duration of acupuncture treatment, which may lead to greater heterogeneity. Meanwhile, the mechanism of acupuncture for the treatment of post-stroke hiccups is not clear.

##### 4.1.2.2 Improvement of sleep quality, diet, and psychological status on post-stroke hiccup

Post-stroke hiccups are commonly associated with sleep disturbances, abnormal dietary habits, and psychological health issues. These factors interact and significantly reduce patients’ quality of life and rehabilitation outcomes. Research has shown that acupuncture treatment can effectively alleviate hiccup symptoms and improve both sleep quality and psychological wellbeing. For example, a study demonstrated that after acupuncture treatment, post-stroke hiccup patients exhibited significant improvements in sleep scores and psychological scores, with enhanced sleep continuity, shorter sleep onset time, and reduced anxiety and depression. Moreover, the improvement in sleep quality was associated with better dietary scores (30). Another study also indicated that acupuncture could relieve hiccups, improve sleep, dietary habits, and psychological health, thus promoting overall patient recovery (31). Acupuncture may alleviate post-stroke hiccups and improve psychological and dietary health by regulating neurotransmitters (32), enhancing cerebral blood flow (31), and balancing the autonomic nervous system (33), thus promoting neuroplasticity and improving sleep quality.

##### 4.1.2.3 Improvement in the incidence of adverse events

With respect to safety, the current evidence base is very limited. Only one systematic review quantitatively analyzed adverse events, suggesting a possible reduction compared with conventional treatment (RR 0.45 [0.16, 1.25]), but the confidence interval crossed unity, and certainty was rated as low. Two additional reviews qualitatively reported fewer adverse events in the acupuncture groups, yet no pooled estimates were available, and most reviews did not systematically assess harms at all. Taken together, these findings indicate that safety data remain sparse and under-reported. Although no serious adverse events were described, the evidence is insufficient to establish a reliable safety profile, and the overall safety of acupuncture for post-stroke hiccup therefore remains uncertain. More rigorous and standardized reporting of adverse events is required in future trials.

As summarized in Table 2, the pooled results indicated relative improvements in clinical efficacy and hiccup symptom scores with acupuncture. While some of these effects reached statistical significance (for example, several reviews reported risk ratios above 1.2), the absolute clinical benefit was modest. In addition, considerable heterogeneity was present in some analyses, and most outcomes were graded as low or very low certainty by GRADE.

### 4.2 Implications for research

Although most studies have found the efficacy of acupuncture in the treatment of post-stroke hiccups to be positive. However, strong evidence for its specific therapeutic course is still lacking. In addition, our study found that acupuncture can alleviate negative emotional states.

In addition, enriched trials with strong participant recruitment should be prioritized to avoid reporting low-quality, inadequate studies, which may improve the ability to detect treatment effects in a population. These details are essential to ensure the reproducibility of studies and comparability of results across studies.

Considering that many systematic reviews on acupuncture for post-stroke hiccups have been conducted over the past 5 years, some of these results have been inconsistent, and the overlap of clinical studies has been relatively high. However, we do not recommend any updated or original systematic reviews. According to the Preferred Reporting Items for Systematic Reviews and Meta-Analyses (PRISMA) (34), it is recommended that a list or rationale for the exclusion of original studies be provided wherever possible, and that reports adhere to established criteria. Reliable systematic reviews are necessary to guide clinical recommendations and decisions. Systematic reviews aim to identify all relevant literature, assess its risk of bias, and evaluate the strength of evidence across trials to provide a comprehensive and in-depth synthesis of the results, rather than just demonstrating efficacy in clinical studies.

### 4.3 Strengths and limitations

Our overview contains pros and cons. We provided a comprehensive summary of all SRs of acupuncture for post-stroke hiccups. We also commented on the clinical significance of these findings. We assessed the strength of outcome evidence. Limitations of the study include the following: first, the overlap of the major randomized controlled trials included in the review may limit interpretation of the results. We attempted to interpret the results of the evidence as fully as possible, but it is important to recognize that the results of the included reviews should complement and support each other. In addition, the small sample sizes of the studies may limit the certainty of conclusions.

## 5 Conclusion

In conclusion, while the reviewed SRs offered valuable insights into the use of acupuncture for post-stroke hiccups, their varied methodologies, reporting standards, and limited evidence quality warrant caution when recommending it as a treatment. Clinical studies should address the critical importance of assessing the efficacy of acupuncture at different intervention times. If acupuncture’s effectiveness in preventing or managing post-stroke hiccups can be more definitively established, this would contribute to clearer and more reliable findings in this vital area of medical research.

## Data Availability

The original contributions presented in the study are included in the article/Supplementary material, further inquiries can be directed to the corresponding author.
