# Supplementary material for: Acupuncture for post-stroke hiccup: an overview of systematic reviews
Source: Front Neurol. 2025 Oct 23;16:1684772. doi: 10.3389/fneur.2025.1684772 (PMC12588814; doi:10.3389/fneur.2025.1684772)
Supplement: Supplementary file 1 [file Table_1.docx]

**Acupuncture for post-stroke hiccup: an overview of systematic reviews**

Xin-Xin Liu^1^, Ying-qi Ma^2^, You-zhu Su^1^, Ling-yao Kong^1^, Chen Shen^1^, Jian-Ping Liu^1,3*^

^1^Centre for Evidence-Based Chinese Medicine, Beijing University of Chinese Medicine, Beijing 100029, China

^2^School of Basic Medical Sciences, Guangzhou university of Chinese medicine, Guangzhou, Guangdong, China.

^3^The National Research Center in Complementary and Alternative Medicine (NAFKAM), Department of Community Medicine, Faculty of Health Science, UiT The Arctic University of Norway, Tromsø, Norway

Correspondence author: Jian-ping Liu^*^, E-mail: liujp@bucm.edu.cn

**Supplementary Material 1**

**Table 1**

Search strategy for PubMed.（**2025-02-20**）

| Search query |
| --- |
| **#1：**Stroke[MeSH Terms] OR Stroke[Title/Abstract] OR cerebral infarction[MeSH Terms] OR cerebral infarction[Title/Abstract] OR intracranial Hemorrhage[MeSH Terms] OR intracranial Hemorrhage[Title/Abstract] OR cerebral hemorrhage[MeSH Terms] OR cerebral hemorrhage[Title/Abstract] OR CVA[Title/Abstract] OR cerebrovascular accident[Title/Abstract] OR encephalorrhagia[Title/Abstract] OR hematencephalon[Title/Abstract]  **#2:** Hiccup[MeSH Terms] OR Hiccup[Title/Abstract] OR Hiccups[Title/Abstract] OR hiccough[Title/Abstract] OR (singultation[Title/Abstract]  **#3：**Acupuncture[MeSH Terms] OR Acupuncture[Title/Abstract] OR Acupressure[MeSH Terms] OR Acupressure[Title/Abstract] OR(electroacupuncture[Title/Abstract] OR laser needle[Title/Abstract] OR scalp acupuncture[Title/Abstract] OR body acupuncture[Title/Abstract] OR nose acupuncture[Title/Abstract] OR needle-embedding therapy[Title/Abstract]  **#4：**#1AND#2  #**5：**#3AND#4  **#6** Limits: Systematic reviews or meta-analysis |
| **命中题录 3** |

**Table 2**

Search strategy for Embase.（**2025-02-20**）

| Search query |
| --- |
| **#1:** 'cerebrovascular accident'/exp OR 'cerebrovascular accident':ab,ti OR 'accident, cerebrovascular':ab,ti OR 'acute cerebrovascular lesion':ab,ti OR 'acute focal cerebral vasculopathy':ab,ti OR 'acute stroke':ab,ti OR 'apoplectic stroke':ab,ti OR 'apoplexia':ab,ti OR 'apoplexy':ab,ti OR 'blood flow disturbance, brain':ab,ti OR 'brain accident':ab,ti OR 'brain attack':ab,ti OR 'brain blood flow disturbance':ab,ti OR 'brain insult':ab,ti OR 'brain insultus':ab,ti OR 'brain vascular accident':ab,ti OR 'cerebral apoplexia':ab,ti OR 'cerebral insult':ab,ti OR 'cerebral stroke':ab,ti OR 'cerebral vascular accident':ab,ti OR 'cerebral vascular insufficiency':ab,ti OR 'cerebro vascular accident':ab,ti OR 'cerebrovascular arrest':ab,ti OR 'cerebrovascular failure':ab,ti OR 'cerebrovascular injury':ab,ti OR 'cerebrovascular insufficiency':ab,ti OR 'cerebrovascular insult':ab,ti OR 'cerebrum vascular accident':ab,ti OR 'cryptogenic stroke':ab,ti OR 'CVA':ab,ti OR 'insultus cerebralis':ab,ti OR 'ischaemic seizure':ab,ti OR 'ischemic seizure':ab,ti OR 'stroke':ab,ti OR 'thrombotic stroke':ab,ti OR 'cerebral infarction'/exp OR 'cerebral infarction':ab,ti OR 'brain cortex infarct':ab,ti OR 'brain cortex infarction':ab,ti OR 'brain infarct':ab,ti OR 'cerebral cortex infarct':ab,ti OR 'cerebral cortex infarction':ab,ti OR 'cerebral infarct':ab,ti OR 'cerebral infarction':ab,ti OR 'cerebrovascular infarct':ab,ti OR 'cerebrovascular infarction':ab,ti OR 'cortical infarct':ab,ti OR 'cortical infarction':ab,ti OR 'hemisphere infarct':ab,ti OR 'hemisphere infarction':ab,ti OR 'hemispheric infarct':ab,ti OR 'hemispheric infarction':ab,ti OR 'infarction, brain':ab,ti OR 'silent brain infarction':ab,ti OR 'brain infarction':ab,ti OR 'brain hemorrhage'/exp OR 'brain hemorrhage':ab,ti OR 'bleeding, corpus callosum':ab,ti OR 'brain bleeding':ab,ti OR 'brain haemorrhage':ab,ti OR 'brain haemorrhage, traumatic':ab,ti OR 'brain hemorrhage, traumatic':ab,ti OR 'brain microhaemorrhage':ab,ti OR 'brain microhemorrhage':ab,ti OR 'brain stem haemorrhage, traumatic':ab,ti OR 'brain stem hemorrhage, traumatic':ab,ti OR 'cerebral haemorrhage':ab,ti OR 'cerebral haemorrhage, traumatic':ab,ti OR 'cerebral hemorrhage':ab,ti OR 'cerebral hemorrhage, traumatic':ab,ti OR 'cerebral microbleed':ab,ti OR 'corpus callosum bleeding':ab,ti OR 'corpus callosum haemorrhage':ab,ti OR 'corpus callosum hemorrhage':ab,ti OR 'encephalorrhagia':ab,ti OR 'haemorrhage, brain':ab,ti OR 'haemorrhage, intracranial':ab,ti OR 'haemorrhagic apoplexy':ab,ti OR 'haemorrhagic stroke':ab,ti OR 'haemorrhagic stroke intracerebral bleeding':ab,ti OR 'hematencephalon':ab,ti OR 'hemorrhage, brain':ab,ti OR 'hemorrhage, intracranial':ab,ti OR 'hemorrhagic apoplexy':ab,ti OR 'hemorrhagic stroke':ab,ti OR 'hemorrhagic stroke intracerebral bleeding':ab,ti OR 'hypertensive intracranial haemorrhage':ab,ti OR 'hypertensive intracranial hemorrhage':ab,ti OR 'intracerebral bleeding':ab,ti OR 'intracerebral haemorrhage':ab,ti OR 'intracerebral hemorrhage':ab,ti OR 'intracortical haemorrhage':ab,ti OR 'intracortical hemorrhage':ab,ti OR 'intracranial bleeding':ab,ti OR 'intracranial haemorrhage':ab,ti OR 'intracranial haemorrhage, hypertensive':ab,ti OR 'intracranial haemorrhage, traumatic':ab,ti OR 'intracranial haemorrhages':ab,ti OR 'intracranial hemorrhage':ab,ti OR 'intracranial hemorrhage, hypertensive':ab,ti OR 'intracranial hemorrhage, traumatic':ab,ti OR 'intracranial hemorrhages':ab,ti OR 'intraventricular haemorrhage':ab,ti OR 'intraventricular hemorrhage':ab,ti OR 'periventricular haemorrhage':ab,ti OR 'periventricular hemorrhage':ab,ti OR 'posterior fossa haemorrhage':ab,ti OR 'posterior fossa hemorrhage':ab,ti OR 'traumatic brain haemorrhage':ab,ti OR 'traumatic brain hemorrhage':ab,ti OR 'traumatic brain stem haemorrhage':ab,ti OR 'traumatic brain stem hemorrhage':ab,ti OR 'traumatic cerebral haemorrhage':ab,ti OR 'traumatic cerebral hemorrhage':ab,ti OR 'traumatic intracranial haemorrhage':ab,ti OR 'traumatic intracranial hemorrhage':ab,ti OR 'brain hemorrhage':ab,ti  **#2：**'hiccup'/exp OR 'hiccup':ab,ti OR 'hiccough':ab,ti OR 'singultus':ab,ti OR 'singultation':ab,ti OR 'phrenospasm':ab,ti OR 'phrenoasm':ab,ti  **#3：**'acupuncture'/exp OR 'acupuncture':ab,ti OR 'acupuncture therapy':ab,ti OR 'electroacupuncture'/exp OR 'electroacupuncture':ab,ti OR 'acupuncture, electric':ab,ti OR 'electric acupuncture':ab,ti OR 'electrical acupoint stimulation':ab,ti OR 'electrical acupuncture':ab,ti OR 'electro-acupuncture':ab,ti OR 'electrode acupuncture':ab,ti OR 'electronic acupuncture':ab,ti OR 'auricular acupuncture'/exp OR 'auricular acupuncture':ab,ti OR 'acupuncture, ear':ab,ti OR 'acupuncture, earlobe':ab,ti OR 'auriculo-acupuncture':ab,ti OR 'auriculoacupuncture':ab,ti OR 'auriculotherapy':ab,ti OR 'ear acupuncture':ab,ti OR 'earlobe acupuncture':ab,ti OR 'laser needle':ab,ti OR 'scalp acupuncture':ab,ti OR 'body acupuncture':ab,ti OR 'nose acupuncture':ab,ti OR 'needle-embedding therapy':ab,ti OR 'Dai Zhen Gao':ab,ti OR 'auricular':ab,ti OR 'auricular point sticking':ab,ti OR 'auricular-plaster':ab,ti OR 'acupressure'/exp OR 'acupressure':ab,ti OR 'plaster on acupuncture points':ab,ti  **#4：** #1 AND #2  **#5：** #3 AND #4  **#6** study types: Systematic reviews or meta-analysis or network meta-analysis  **#7** publications types:Review |
| **命中题录 30** |

**Table 3**

Search strategy for WOS.（**2025-02-20**）

| Search query |
| --- |
| **#1:** TS=("stroke" or "CVA" or "cerebrovascular accident" or "cerebral infarction")  **#2:** TS=("Hiccups" or "hiccoughs" or "singulation" or "singulation" or "phrenospasm")  **#3:** TS=("acupuncture" or "electroacupuncture" or "auricular acupuncture" or "scalp acupuncture" or "body acupuncture" or "nose acupuncture" )  **#4：** #1AND #2  **#5：** #3 AND #4  **#6** Review article |
| **命中题录 5** |

**Table 4**

Search strategy for Cochrane.（**2025-02-20**）

| Search query |
| --- |
| **#1** MeSH descriptor: [Acupuncture] explode all trees  **#2** (Pharmacopuncture):ti,ab,kw  **#3** MeSH descriptor: [Electroacupuncture] explode all trees  **#4** (auricular acupuncture):ti,ab,kw  **#5** MeSH descriptor: [Acupuncture, Ear] explode all trees  **#6** (Ear Acupuncture):ti,ab,kw  **#7** (Auricular Acupuncture):ti,ab,kw  **#8** (Acupuncture, Auricular):ti,ab,kw  **#9** (Acupunctures, Auricular):ti,ab,kw  **#10** (Auricular Acupunctures):ti,ab,kw  **#11** (Acupunctures, Ear):ti,ab,kw  **#12** (Ear Acupunctures):ti,ab,kw  **#13** (laser needle):ti,ab,kw  **#14** (scalp acupuncture):ti,ab,kw  **#15** (body acupuncture):ti,ab,kw  **#16** (nose acupuncture):ti,ab,kw  **#17** (needle-embedding therapy):ti,ab,kw  **#18** (Dai Zhen Gao):ti,ab,kw  **#19** (auricular):ti,ab,kw  **#20** (auricular point sticking):ti,ab,kw  **#21** (auricular-plaster):ti,ab,kw  **#22** MeSH descriptor: [Acupressure] explode all trees  **#23** (plaster on acupuncture points):ti,ab,kw  **#24** MeSH descriptor: [Stroke] explode all trees  **#25** (Cerebral Strokes):ti,ab,kw  **#26** (Cerebral Stroke):ti,ab,kw  **#27** (Vascular Accident, Brain):ti,ab,kw  **#28** (Strokes; Apoplexy, Cerebrovascular):ti,ab,kw  **#29** (Apoplexy):ti,ab,kw  **#30** (Cerebrovascular Apoplexy):ti,ab,kw  **#31** (Cerebrovascular Stroke):ti,ab,kw  **#32** (Stroke, Cerebrovascular):ti,ab,kw  **#33** (Cerebrovascular Accident):ti,ab,kw  **#34** (Brain Vascular Accident):ti,ab,kw  **#35** (Cerebrovascular Accidents):ti,ab,kw  **#36** (Brain Vascular Accidents):ti,ab,kw  **#37** (Cerebrovascular Strokes):ti,ab,kw  **#38** (Stroke, Cerebral):ti,ab,kw  **#39** (CVAs (Cerebrovascular Accident)):ti,ab,kw  **#40** (Strokes, Cerebrovascular):ti,ab,kw  **#41** (CVA (Cerebrovascular Accident)):ti,ab,kw  **#42** (Vascular Accidents, Brain):ti,ab,kw  **#43** (Strokes, Cerebral):ti,ab,kw  **#44** (Cerebrovascular Accidents, Acute):ti,ab,kw  **#45** (Cerebrovascular Accident, Acute):ti,ab,kw  **#46** (Acute Strokes):ti,ab,kw  **#47** (Strokes, Acute):ti,ab,kw  **#48** (Acute Stroke):ti,ab,kw  **#49** (Acute Cerebrovascular Accident):ti,ab,kw  **#50** (Acute Cerebrovascular Accidents):ti,ab,kw  **#51** (Stroke, Acute):ti,ab,kw  **#52** (cerebral infarction):ti,ab,kw  **#53** (intracranial Hemorrhage):ti,ab,kw  **#54** (cerebral hemorrhage):ti,ab,kw  **#55** (encephalorrhagia):ti,ab,kw  **#56** (hematencephalon):ti,ab,kw  **#57** (hiccups):ti,ab,kw  **#58** MeSH descriptor: [Hiccup] explode all trees  **#59** (intractable hiccup):ti,ab,kw  **#60** (hiccough):ti,ab,kw  **#61** (singultation):ti,ab,kw  **#62** (obstinate singultus):ti,ab,kw  **#63** (phrenospasm):ti,ab,kw  **#64** (phrenoasm):ti,ab,kw  **#65** #1 OR #2 OR #3 OR #4 OR #5 OR #6 OR #7 OR #8 OR #9 OR #10 OR #11 OR #12 OR #13 OR #14 OR #15 OR #16 OR #17 OR #18 OR #19 OR #20 OR #21 OR #22 OR #23  **#66** #24 OR #25 OR #26 OR #27 OR #28 OR #29 OR #30 OR #31 OR #32 OR #33 OR #34 OR #35 OR #36 OR #37 OR #38 OR #39 OR #40 OR #41 OR #42 OR #43 OR #44 OR #45 OR #46 OR #47 OR #48 OR #49 OR #50 OR #51 OR #52 OR #53 OR #54 OR #55 OR #56  **#67** #57 OR #58 OR #59 OR #60 OR #61 OR #62 OR #63 OR #64  **#68** #66 AND #67  **#69** #65 AND #68  **#70** Cochrane Reviews |
| **命中题录 1** |

**Table 5**

Search strategy for CNKI.（**2025-02-20**）

| Search query |
| --- |
| **#1：**((SU=(卒中) OR SU=(中风) OR SU=(脑梗死) OR SU=(脑出血) OR SU=(脑血管意外) OR TKA=(卒中) OR TKA=(中风) OR TKA=(脑梗死) OR TKA=(脑出血) OR TKA=(脑血管意外)))  **#2：**((SU=(呃逆) OR SU=(膈肌痉挛) OR TKA=(呃逆) OR TKA=(膈肌痉挛)))  **#3：**(( SU=(针灸) OR SU=(针刺) OR SU=(电针) OR SU=(耳针) OR SU=(极光针) OR SU=(针刀) OR SU=(滚针) OR SU=(头针) OR SU=(体针) OR SU=(腹针) OR SU=(鼻针) OR SU=(脚踝针) OR SU=(埋针) OR SU=(埋线) OR SU=(耳穴) OR SU=(耳穴贴压) OR SU=(耳压) OR SU=(穴位按压) OR TKA=(针灸) OR TKA=(针刺) OR TKA=(电针) OR TKA=(耳针) OR TKA=(极光针) OR TKA=(针刀) OR TKA=(滚针) OR TKA=(头针) OR TKA=(体针) OR TKA=(腹针) OR TKA=(鼻针) OR TKA=(脚踝针) OR TKA=(埋针) OR TKA=(埋线) OR TKA=(耳穴) OR TKA=(耳穴贴压) OR TKA=(耳压) OR TKA=(穴位按压))))  **#4：**(((TKA=(系统综述) OR TKA=(系统评价) OR TKA=(荟萃分析) OR TKA=(meta分析))  **#5：**#1AND#2  **#6：**#3AND#5AND#4 |
| **命中题录 7** |

**Table 6**

Search strategy for WANFANG.（**2025-02-20**）

| Search query |
| --- |
| **#1：**主题:(卒中 OR 中风 OR 脑梗死 OR 脑出血 OR 脑血管意外)  **#2：**主题:(呃逆 OR 膈肌痉挛)  **#3：**主题:(针灸 OR 针刺 OR 电针 OR 耳针 OR 极光针 OR 针刀 OR 滚针 OR 头针 OR 体针 OR 腹针 OR 鼻针 OR 脚踝针 OR 埋针 OR 埋线 OR 代针膏 OR 埋线 OR 耳穴 OR 耳穴贴压 OR 耳压 OR 穴位按压)  **#4：**主题:(系统综述 OR 系统评价 OR 荟萃分析 OR meta分析)  **#5：**#1AND#2  **#6：**#3AND#5AND#4 |
| **命中题录 13** |

**Table 7**

Search strategy for VIP.（**2025-02-20**）

| Search query |
| --- |
| **#1：**(M=(卒中 OR 中风 OR 脑梗死 OR 脑出血 OR 脑血管意外) OR R=(卒中 OR 中风 OR 脑梗死 OR 脑出血 OR 脑血管意外))  **#2：**(M=(呃逆 OR 膈肌痉挛) OR R=(呃逆 OR 膈肌痉挛))  **#3：**((M=(针灸 OR 针刺 OR 电针 OR 耳针 OR 极光针 OR 针刀 OR 滚针 OR 头针 OR 体针 OR 腹针 OR 鼻针 OR 脚踝针 OR 埋针 OR 埋线 OR 代针膏 OR 埋线 OR 耳穴 OR 耳穴贴压 OR 耳压 OR 穴位按压)) OR (R=(针灸 OR 针刺 OR 电针 OR 耳针 OR 极光针 OR 针刀 OR 滚针 OR 头针 OR 体针 OR 腹针 OR 鼻针 OR 脚踝针 OR 埋针 OR 埋线 OR 代针膏 OR 埋线 OR 耳穴 OR 耳穴贴压 OR 耳压 OR 穴位按压)))  **#4：**(M=(系统综述 OR 系统评价 OR 荟萃分析 OR meta分析) OR R=(系统综述 OR 系统评价 OR 荟萃分析 OR meta分析))  **#5：**#1AND#2  **#6：**#3AND#5AND#4 |
| **命中题录 14** |

**Table 8**

Search strategy for VIP.（**2025-02-20**）

| Search query |
| --- |
| **#1：**("呃逆"[常用字段:智能] OR "膈肌痉挛"[常用字段:智能])  **#2：**("卒中"[常用字段:智能] OR "中风"[常用字段:智能] OR "脑梗死"[常用字段:智能] OR "脑出血"[常用字段:智能] OR "脑血管意外"[常用字段:智能])  **#3：**("针灸"[常用字段:智能] OR "针刺"[常用字段:智能] OR "电针"[常用字段:智能] OR "耳针"[常用字段:智能] OR "极光针"[常用字段:智能] OR "针刀"[常用字段:智能] OR "滚针"[常用字段:智能] OR "头针"[常用字段:智能] OR "腹针"[常用字段:智能] OR "鼻针"[常用字段:智能] OR "脚踝针"[常用字段:智能] OR "埋针"[常用字段:智能] OR "埋线"[常用字段:智能] OR "代针膏"[常用字段:智能] OR "耳穴"[常用字段:智能] OR "耳穴贴压"[常用字段:智能] OR "耳压"[常用字段:智能] OR "穴位按压"[常用字段:智能])  **#4：**("系统综述"[标题:智能] OR "系统评价"[标题:智能] OR "荟萃分析"[标题:智能] OR "meta分析"[标题:智能])  **#5：**#1AND#2  **#6：**#3AND#5AND#4 |
| **命中题录 6** |

**Supplementary Material 2**

| **Primary studies** | **Included 4 systematic reviews on acupuncture for Hiccup after stroke( Hiccup symptom scores)**  **(32.50%/10.00%,N=26,c=4,r=20)** | | | |
| --- | --- | --- | --- | --- |
| **Study ID** | Wang 2023 | Zhang 2022 | Zhao 2021 | Liu 2015 |
| Chen S2017 | √ |  |  |  |
| Chi XF2019 | √ |  |  |  |
| Cui MJ2021 | √ |  |  |  |
| Guo ZG2018 | √ |  |  |  |
| Li CL2019 |  |  | √ |  |
| Liu L2019 |  |  | √ |  |
| Qiu WZ2017 |  | √ |  |  |
| Wang JH2019 | √ |  |  |  |
| Wang L2020 | √ |  |  |  |
| Wang ZG2019 | √ |  |  |  |
| Xu XXa2012 |  |  |  | √ |
| Xu XXb2012 |  |  |  | √ |
| Yan XR2011 |  | √ |  | √ |
| Yan XR2012 |  | √ |  | √ |
| Yang J2015 | √ | √ |  |  |
| Yang LL2019 | √ |  |  |  |
| Yang QT2017 | √ | √ |  |  |
| Zhang J2021 | √ |  |  |  |
| Zhang MY2015 |  | √ | √ |  |
| Zheng DS2016 | √ | √ |  |  |

| **Primary studies** | **Included 10 systematic reviews on acupuncture for Hiccup after stroke(Clinical efficiency)**  **(16.90%/7.67%,N=93,c=10,r=55)** | | | | | | | | | |
| --- | --- | --- | --- | --- | --- | --- | --- | --- | --- | --- |
| **Study ID** | Wang 2023 | Zhang  2022 | Zhao 2021 | Chen  2020 | Zhang 2019 | Yue 2017 | Liu 2015 | He  2013 | Zhu  2011 | Lin  2010 |
| Bai YM2004 |  |  |  |  |  |  |  | √ |  |  |
| Cao GJ2010 |  | √ |  |  | √ |  |  |  |  |  |
| Cao JJ2001 |  |  |  |  |  |  | √ |  |  |  |
| Chen S2017 | √ |  |  | √ |  |  |  |  |  |  |
| Chen YF2009 |  |  |  |  |  |  | √ |  |  |  |
| Chi XF2019 | √ |  |  |  |  |  |  |  |  |  |
| Cui MJ2021 | √ |  |  |  |  |  |  |  |  |  |
| Fu K2008 |  |  |  |  |  |  |  | √ |  |  |
| Guo HY2020 | √ |  |  |  |  |  |  |  |  |  |
| Guo ZG2018 | √ |  |  |  |  |  |  |  |  |  |
| Jiang HX2010 |  |  |  |  |  | √ |  |  |  |  |
| Jiang W2010 |  |  |  |  |  |  | √ |  |  |  |
| Li CL2019 |  |  | √ |  |  |  |  |  |  |  |
| Li L2013 |  | √ |  |  | √ |  | √ |  |  |  |
| Lin CG2015 |  |  |  |  |  |  | √ | √ |  | √ |
| Liu CY2009 | √ | √ |  |  |  |  |  | √ |  |  |
| Liu L2019 |  |  | √ |  |  |  |  |  |  |  |
| Liu LM2012 | √ |  |  |  |  |  |  |  |  |  |
| Liu Q2015 |  | √ |  |  |  |  |  |  |  |  |
| Liu WX2013 |  |  |  |  |  |  | √ |  |  |  |
| Liu YG2021 | √ |  |  |  |  |  |  |  |  |  |
| Liu YM2016 |  | √ |  |  |  |  |  |  |  |  |
| Liu YQ2015 |  |  | √ |  |  |  |  |  |  |  |
| Qiu WZ2017 |  | √ |  | √ |  |  |  |  |  |  |
| Qu M2010 |  |  |  |  |  |  |  | √ |  |  |
| Ren JJ2009 |  |  |  |  |  |  |  |  | √ |  |
| Ruan Q2011 |  |  |  |  |  |  |  | √ |  |  |
| Su CM2007 | √ | √ |  | √ | √ |  | √ | √ |  |  |
| Sun Y2009 |  | √ |  |  | √ |  | √ | √ |  |  |
| Tan Y2017 |  |  | √ |  |  |  |  |  |  |  |
| Wang B2017 |  | √ |  | √ |  |  |  |  |  |  |
| Wang L2020 | √ |  |  |  |  |  |  |  |  |  |
| Wang Y2014 |  |  |  |  |  |  | √ |  |  |  |
| Wang ZG2019 | √ |  |  |  |  |  |  |  |  |  |
| Wei Q2014 |  | √ |  |  |  | √ | √ |  |  |  |
| Xi MJ2008 |  |  |  |  | √ |  | √ |  |  |  |
| Xiong T2005 |  |  |  |  |  |  |  |  |  | √ |
| Xu SJ2015 |  | √ |  |  |  |  |  |  |  |  |
| Xu XXa2012 |  |  |  |  |  |  | √ |  |  |  |
| Xu XXb2012 |  |  |  |  |  |  | √ |  |  |  |
| Yan XR2011 |  | √ |  |  |  |  | √ |  |  |  |
| Yan XR2012 |  | √ |  |  |  | √ | √ |  |  |  |
| Yan Y2004 |  |  |  |  |  |  |  |  | √ | √ |
| Yang J2015 | √ | √ |  |  |  |  |  |  |  |  |
| Yang LL2019 | √ |  |  |  |  |  |  |  |  |  |
| Yang QT2017 | √ | √ |  |  | √ |  |  |  |  |  |
| Zhang AJ2009 |  |  |  |  |  |  |  | √ |  |  |
| Zhang HL2020 | √ |  |  |  |  |  |  |  |  |  |
| Zhang J2021 | √ |  |  |  |  |  |  |  |  |  |
| Zhang M2012 |  |  |  |  |  |  | √ |  |  |  |
| Zhang MY2015 |  | √ | √ |  | √ |  |  |  |  |  |
| Zhang XT2006 |  | √ |  |  | √ | √ | √ |  |  | √ |
| Zhu P2008 |  |  |  |  |  |  | √ |  |  |  |
| Zhu XW2017 |  | √ |  | √ | √ |  |  |  |  |  |
| Zong F2009 |  |  |  |  |  | √ | √ |  | √ |  |

| **Primary studies** | **Included 3 systematic reviews on acupuncture for Hiccup after stroke( Dietary scores)**  **(37.03%/5.55%,N=10,c=3,r=9)** | | |
| --- | --- | --- | --- |
| **Study ID** | Zhang 2022 | Zhao 2021 | Chen 2020 |
| Cao YH2016 |  | √ |  |
| Fan JC2018 |  | √ |  |
| Huang YT2016 |  |  | √ |
| Li Q2015 | √ |  |  |
| Qiu WZ2017 | √ |  | √ |
| Wang B2017 | √ |  | √ |
| Wang Y2012 |  | √ |  |
| Zhang MZ2018 |  |  | √ |

| **Primary studies** | **Included 3 systematic reviews on acupuncture for Hiccup after stroke( Psychology scores)**  **(40.74%/11.11%,N=11,c=3,r=9)** | | |
| --- | --- | --- | --- |
| **Study ID** | Zhang 2022 | Zhao 2021 | Chen 2020 |
| Cao YH2016 |  | √ |  |
| Fan JC2018 |  | √ |  |
| Huang YT2016 |  |  | √ |
| Li Q2015 | √ |  |  |
| Li ZW2013 |  |  | √ |
| Qiu WZ2017 | √ |  | √ |
| Wang B2017 | √ |  | √ |
| Wang Y2012 |  | √ |  |
| Zhang MZ2018 |  |  | √ |

| **Primary studies** | **Included 3 systematic reviews on acupuncture for Hiccup after stroke( Sleep scores)**  **(40.74%/11.11%,N=11,c=3,r=9)** | | |
| --- | --- | --- | --- |
| **Study ID** | Zhang 2022 | Zhao 2021 | Chen 2020 |
| Cao YH2016 |  | √ |  |
| Fan JC2018 |  | √ |  |
| Huang YT2016 |  |  | √ |
| Li Q2015 | √ |  |  |
| Li ZW2013 |  |  | √ |
| Qiu WZ2017 | √ |  | √ |
| Wang B2017 | √ |  | √ |
| Wang Y2012 |  | √ |  |
| Zhang MZ2018 |  |  | √ |
